# Cross-neutralization ability of anti-MERS-CoV monoclonal antibodies against a variety of merbecoviruses

**DOI:** 10.3389/fmicb.2025.1593095

**Published:** 2025-07-16

**Authors:** Lin Pan, Yu Kaku, Jarel Elgin Tolentino, Yusuke Kosugi, Kei Sato

**Affiliations:** ^1^Division of Systems Virology, Department of Microbiology and Immunology, The Institute of Medical Science, The University of Tokyo, Tokyo, Japan; ^2^Department of Computational Biology and Medical Sciences, Graduate School of Frontier Sciences, The University of Tokyo, Kashiwa, Japan; ^3^Graduate School of Medicine, The University of Tokyo, Tokyo, Japan; ^4^International Research Center for Infectious Diseases, The Institute of Medical Science, The University of Tokyo, Tokyo, Japan; ^5^International Vaccine Design Center, The Institute of Medical Science, The University of Tokyo, Tokyo, Japan; ^6^Collaboration Unit for Infection, Joint Research Center for Human Retrovirus Infection, Kumamoto University, Kumamoto, Japan; ^7^MRC-University of Glasgow Centre for Virus Research, Glasgow, United Kingdom

**Keywords:** merbecovirus, MERS-CoV, neutralizing antibody, spillover, outbreak, pandemic

## Abstract

In the 21st century, three severe human coronavirus infections have occurred. One of them is the Middle East respiratory syndrome coronavirus (MERS-CoV), a merbecovirus belonging to the family *Coronaviridae*, is a human pathogenic coronavirus first detected in 2012. Several monoclonal antibodies (mAbs) have been developed for both therapeutics and prevention of MERS-CoV infection. However, the extent to which these anti-MERS-CoV antibodies neutralize other merbecoviruses remains unclear. Here, we evaluated the cross-neutralization ability of ten anti-MERS-CoV mAbs against the pseudoviruses with the spike proteins of five merbecoviruses known to bind to dipeptidyl peptidase 4 (DPP4): three clades of MERS-CoV, a bat-derived merbecovirus (BtCoV-422) and a pangolin-derived merbecovirus (MjHKU4r-CoV). We show that all eight mAbs targeting the receptor-binding domain (RBD) potently neutralize all MERS-CoV clades, but not BtCoV-422 and MjHKU4r-CoV. Of these, the neutralization potency of one mAb, m336, against the MERS-CoV clade B declined due to the V530L substitution detected in certain isolates during the 2015 outbreak in South Korea. On the other hand, although BtCoV-422 was neutralized by the two non-RBD mAbs, 7D10 (targeting the N-terminal domain) and G4 (targeting the S2 subunit), MjHKU4r-CoV found to be resistant. Our findings suggest that combining multiple mAbs targeting different epitopes could be a promising strategy for prevention of future outbreaks caused by novel pathogenic merbecoviruses.

## Introduction

Betacoronaviruses are the viruses belonging to the family *Coronaviridae*. In the last decades, three highly pathogenic and contagious betacoronaviruses have emerged in the human population: severe acute respiratory syndrome coronavirus (SARS-CoV) in 2002, Middle East respiratory syndrome coronavirus (MERS-CoV) in 2012, and SARS-CoV-2 in 2019 (Neumann and Kawaoka, 2023). Among these severe disease-causing coronaviruses (CoVs), MERS-CoV has the highest case-fatality rate at ~35% (WHO, 2024), and is the only documented member of the subgenus merbecovirus to infect humans (Tolentino et al., 2024).

MERS-CoV infections in humans often originate from dromedary camels (*Camelus dromedarius*), with most cases occurring in the Arabian Peninsula (WHO, 2024; Hassan et al., 2025; Meyer et al., 2016; Zaki et al., 2012). MERS-CoV outbreak can also be amplified by limited person-to-person transmission, especially during patient care or treatment (Cotten et al., 2014; Kim et al., 2017). Travel-associated cases also triggered MERS outbreaks in countries outside of the Arabian Peninsula (Su et al., 2016; Memish et al., 2013). Of these, the largest outbreak occurred in South Korea in 2015 and was linked to healthcare, resulting in 186 confirmed cases and 38 reported deaths (Oh et al., 2018; WHO, 2015).

MERS-CoV is phylogenetically classified into three major clades: A, B, and C (Chu et al., 2018; Kiambi et al., 2018; Farrag et al., 2021). The first recorded outbreak in Saudi Arabia in 2012 was caused by clade A (Zhou et al., 2021), for which there have been no recent reports of circulation (Hassan et al., 2025; Ngere et al., 2022; Yusof et al., 2017; Reusken et al., 2014; Azhar et al., 2023). Succeeding major outbreaks, including the 2015 outbreak in South Korea, were attributed to clade B (Park et al., 2015; Kim et al., 2020). Clade C circulates in camels in Africa without human infection cases reported (Chu et al., 2018).

Other merbecoviruses, such as bat MERS-related CoVs (MERSr-CoVs), HKU4-related CoVs (HKU4r-CoVs), HKU5-related CoVs (HKU5r-CoVs) and Hedgehog coronavirus-1 (Hedgehog-CoV-1), have been identified in various mammals (Xiong et al., 2022; Tolentino et al., 2024; Han et al., 2023; Li et al., 2021). MERSr-CoVs were mostly identified in bats (Tolentino et al., 2024). HKU4r-CoVs were detected in *Tylonycteris* bat species and pangolins (Chen et al., 2023; Woo et al., 2006), while HKU5r-CoVs were primarily identified in *Pipistrellus abramus* (Woo et al., 2006) but were recently detected in minks (*Neogale vison*) (Zhao et al., 2024). Hedgehog-CoV-1 was detected in hedgehogs (*Erinaceus* species) (Pomorska-Mol et al., 2022; Lau et al., 2019). Not all but some of these merbecoviruses are known to bind to the cellular receptor DPP4 expressed on host cells to initiate infection in the same way as MERS-CoV (Tolentino et al., 2024; Raj et al., 2013).

The DPP4 recognition by the merbecoviruses is mediated by viral spike (S) protein, specifically by the receptor-binding domain (RBD) in the S1 subunit of S protein. Thus, blocking RBD-DPP4 interaction is the main neutralization mechanism of most MERS-CoV monoclonal antibodies (mAbs) (Tai et al., 2023; Tse et al., 2023). Other anti-MERS-CoV mAbs are reported to neutralize the virus by binding to other epitopes on the S protein than the RBD leading to impaired viral entry (Pallesen et al., 2017; Zhou et al., 2019; Wang N. et al., 2019). The epitopes include the followings: the N-terminal domain (NTD) in the S1 subunit, which supports viral entry and immune interactions; and the other functional subunits, S2, which triggers the fusion of viral and cellular membranes (Pallesen et al., 2017; Gui et al., 2017; Kirchdoerfer et al., 2016; Walls et al., 2016; Yuan et al., 2017).

Several anti-MERS-CoV mAbs have advanced to preclinical or clinical evaluation as candidates for both prevention and treatment of MERS-CoV infections (Sivapalasingam et al., 2022; Pascal et al., 2015; Wang et al., 2018; Corti et al., 2015; Chen et al., 2017). It should be noted that the antiviral activity of almost all anti-MERS-CoV mAbs has been evaluated using the prototype MERS-CoV, strain HCoV-EMC/2012, which is categorized in clade A (Goo et al., 2020). However, as mentioned above, clade A is currently not circulating in the human population. Nevertheless, the cross-neutralization activity of anti-MERS-CoV mAbs against other MERS-CoV clades remained elusive. Moreover, it has been revealed that at the S proteins of at least two merbecoviruses, BtCoV/Ii/GD/2014–422 (BtCoV-422; a MERSr-CoV) (Tse et al., 2023) and MjHKU4r-CoV (MjHKU4r-CoV; an HKU4r-CoV) (Chen et al., 2023), both detected in a bat and a pangolin respectively, can use human DPP4 for infection. These observations suggest that merbecoviruses capable of infecting humans exist in the wild and that the spillover of these viruses may pose a risk to the human population. Therefore, understanding the cross-neutralization ability of anti-MERS-CoV mAbs across different MERS-CoV clades and other merbecoviruses could provide valuable insights for the development of broad-spectrum therapeutics and countermeasures.

In this study, we evaluated the cross-neutralization ability of ten anti-MERS-CoV mAbs against a diverse panel of pseudoviruses representing the S proteins from major MERS-CoV clades (A, B, and C) and other DPP4-dependent merbecoviruses, including bat CoV BtCoV-422 and pangolin CoV MjHKU4r-CoV. We provide insight into the development of broad-spectrum therapeutics for global health preparedness against pre-emergent merbecoviruses that have the potential to cause diseases in humans in the future.

## Results

### Preparation of the pseudoviruses with the S proteins of three MERS-CoV strains and two merbecoviruses from a bat and a pangolin

**Figure 1:**
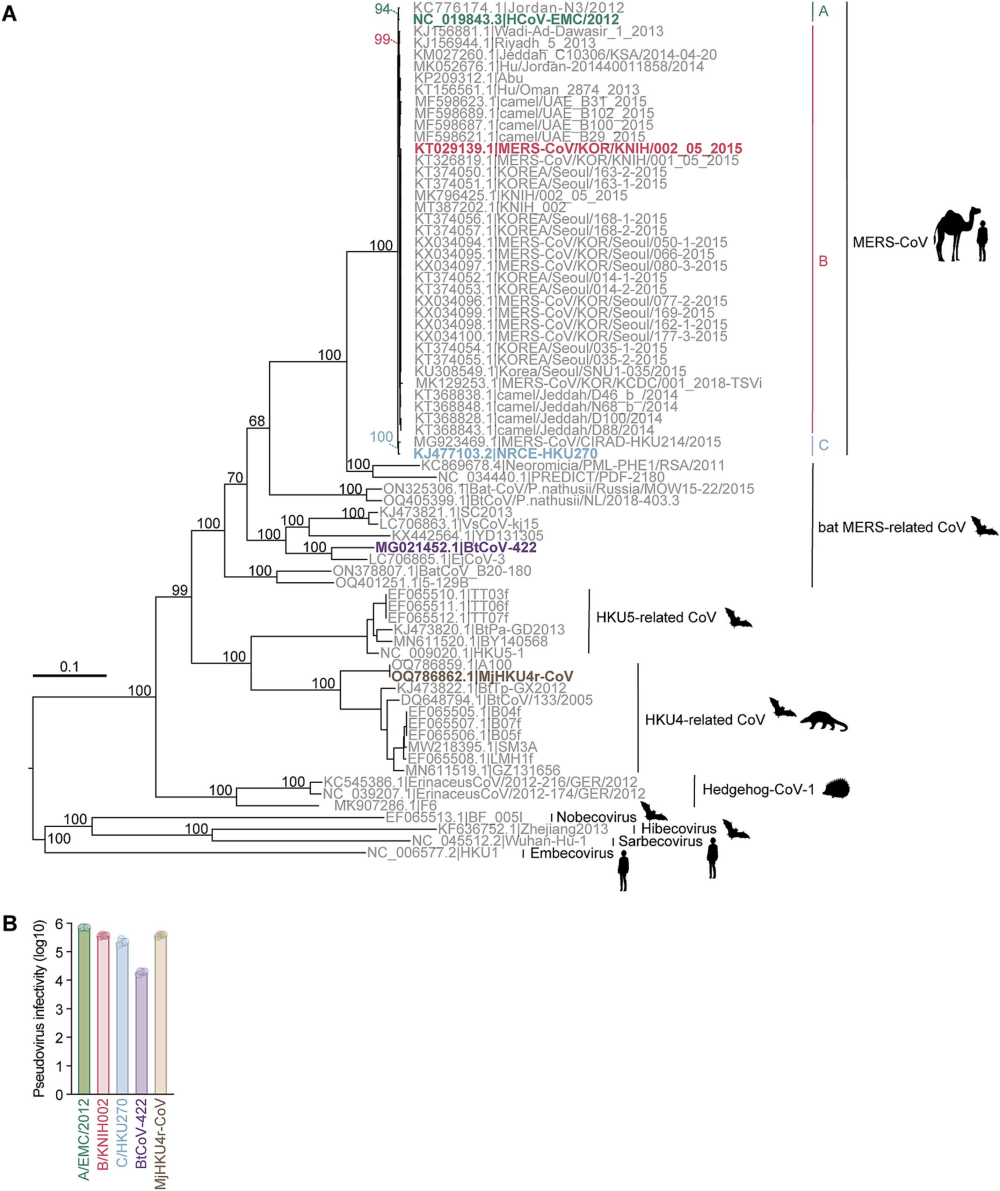
Phylogenetic analysis and infectivity of merbecoviruses having high usage of human DPP4. **(A)** Maximum likelihood-based tree of the merbecovirus complete genome sequences. The highlighted name with animal silhouettes represents the DPP4-using viruses used for experiment. Four representative betacoronaviruses were used as the outgroup. **(B)** Infectivity assay. HOS-human DPP4/TMPRSS2 cells were infected with pseudoviruses bearing each S. The amount of input virus was adjusted to match the amount of HIV-1 p24 capsid protein. Assays were performed in quadruplicate. The presented data are expressed as the average ± SD of relative light unit (RLU). Each dot indicates the result of an individual replicate.

To understand the evolutionary relationship among merbecoviruses, particularly those utilizing DPP4 as their viral receptor, we conducted a phylogenetic analysis using the complete merbecovirus genome sequences (Figure 1A). Following the classification scheme outlined in our previous study (Tolentino et al., 2024), we selected five coronaviruses from each DPP4-using merbecovirus group as representatives for infectivity and neutralization experiments. These includes three MERS-CoV strains: HCoV-EMC/2012 from clade A (A/EMC/2012) (van Boheemen et al., 2012), MERS-CoV/KOR/KNIH/002_05_2015 from clade B (B/KNIH002) (Kim et al., 2015), and NRCE-HKU270 from clade C (C/HKU270) (Chu et al., 2014), as well as MERSr-CoV BtCoV-422 (Tse et al., 2023) and HKU4r-CoV MjHKU4r-CoV (Chen et al., 2023) (Figure 1A). We prepared the lentivirus-based pseudoviruses bearing the S proteins of these five merbecoviruses. To test the possibility of using human DPP4 for infection, we used the human HOS cell line stably expressing human DPP4 and TMPRSS2 (HOS-hDPP4/TMPRSS2) (Barabona et al., 2024) as target cells for pseudovirus infection. As shown in Figure 1B, HOS-hDPP4/TMPRSS2 cells are susceptible to the pseudoviruses with the five merbecovirus S proteins, suggesting that these five merbecoviruses can use human DPP4 as its infection receptor.

### Cross-reactivity of MERS-CoV S RBD-targeting mAbs against three MERS-CoV strains and two merbecoviruses from a bat and a pangolin

**Figure 2:**
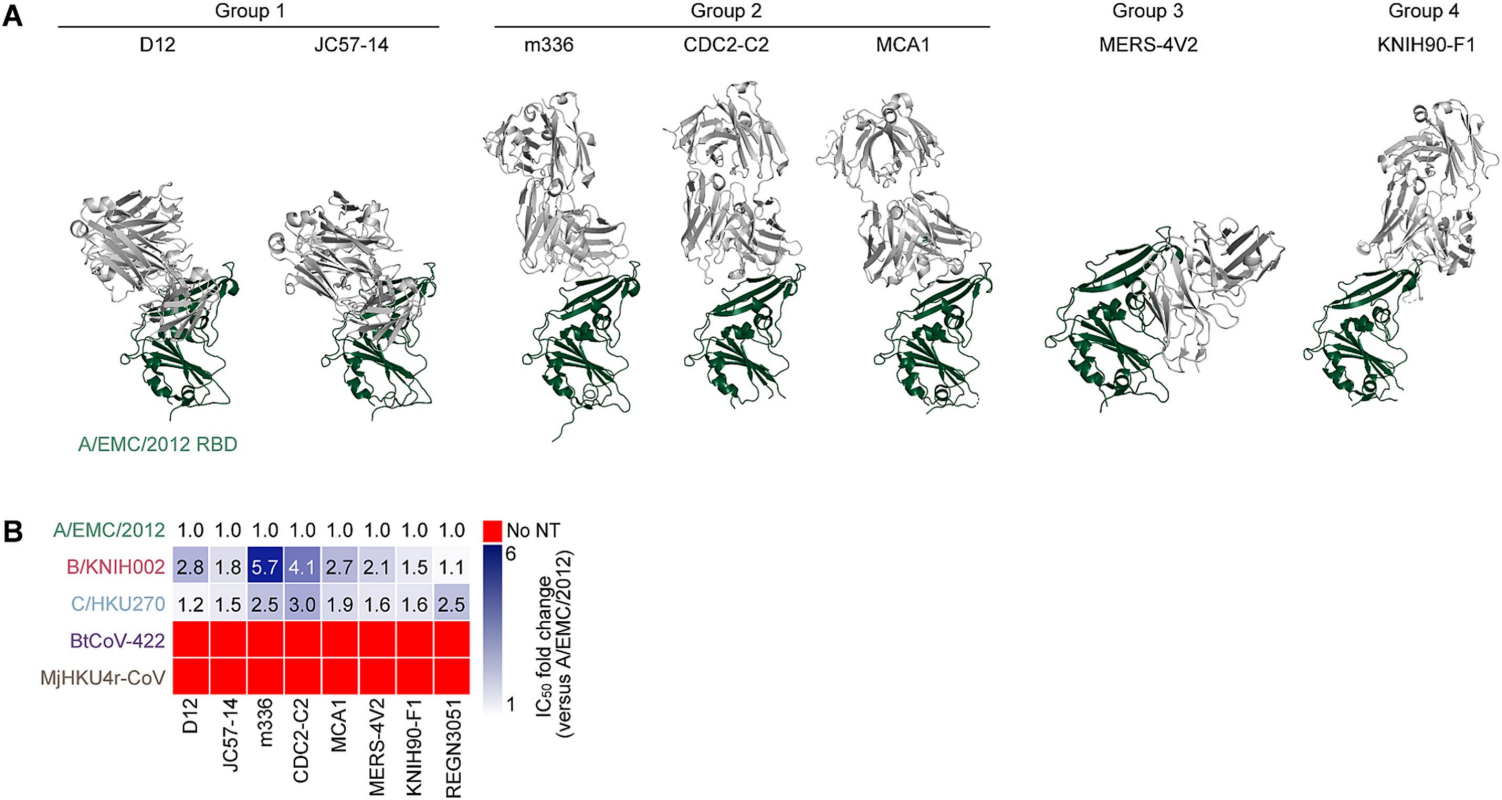
Binding modes and cross-reactivity of MERS-CoV S RBD-targeting mAbs. **(A)** Types of MERS-CoV neutralizing antibodies targeting RBD. Antibodies are classified into three groups and shown as cartoon representation. The A/EMC/2012 RBD is colored in green and antibodies in gray. Group 1 antibodies include D12 (PDB ID: 4ZPT) and JC57-14 (PDB ID: 6C6Y). Group 2 antibodies include m336 (PDB ID: 4XAK), CDC2-C2 (PDB ID: 6C6Z) and MCA1 (PDB ID: 5GMQ), Group 3 antibody includes MERS-4 V2 (PDB ID: 5YY5), Group 4 antibody includes KNIH90-F1 (PDB ID: 4ZPT). **(B)** Neutralization breadth of RBD-targeting mAbs. The heatmap displays the fold change in 50% inhibitory concentration (IC_50_) values, divided by an IC_50_ against A/EMC/2012. The color scheme shows the IC_50_ fold change against 3 clades of MERS-CoVs in addition to BtCoV-422 and MjHKU4r-CoV as the intensity of the blue color in the heatmap. The darkest blue indicates either the fold change more than 6 or the mAb failed to reach IC_50_ at the highest concentration tested. The red indicates no neutralization (no NT).

To investigate the sensitivity of these five merbecoviruses to anti-MERS-CoV mAbs, we selected thirteen MERS-CoV S-targeting mAbs that were previously isolated or characterized including 2 mAbs (REGN3048 and REGN3051), which are the ones in clinical trials for MERS and further in clinical use for SARS-CoV-2 infection (Pascal et al., 2015; de Wit et al., 2018; Gao et al., 2023) and other eleven mAbs selected according to the following four criteria: (1) epitopes are determined by structural analysis; (2) crystal structures of a fragment antigen-binding and a target antigen are available; (3) potent MERS-CoV clade A neutralization activity; and (4) well referred in several previous reports (Wang et al., 2018; Chen et al., 2017; Wang et al., 2015; Houser et al., 2016; Li et al., 2015; Agrawal et al., 2016; van Doremalen et al., 2017; Ying et al., 2015; Niu et al., 2018; Jiang et al., 2014; Zhang et al., 2018; Jang et al., 2022; Choi et al., 2020; Failayev et al., 2024; Zhou et al., 2019; Wang C. et al., 2019). However, three out of the eleven mAb recombinants, MERS-27, 4C2 and REGN3048, were excluded from this study because their neutralization activity against A/EMC/2012 was lost after recombination. Therefore, we used ten mAbs including eight RBD-targeting mAbs and two non-RBD-targeting mAbs in this study. The eight RBD-targeting mAbs without REGN3051, whose epitope was not detected, were categorized into four epitope groups based on their epitopes on RBD: group 1 (D12 and JC57-14), group 2 (m336, MCA1, and CDC2-C2), group 3 (MERS-4 V2), group 4 (KNIH90-F1) (Figure 2A) (Jang et al., 2022; Han et al., 2018). We performed neutralization assays using these mAbs and pseudoviruses. As shown in Figure 2B and Table 1, seven out of the eight MERS-CoV S RBD-targeting mAbs efficiently neutralized all three MERS-CoV strains. However, m336 showed the most weakened neutralization against B/KNIH002 compared to A/EMC/2012 (5.7-fold). In contrast to the three MERS-CoVs, bat CoV BtCoV-422 and pangolin CoV MjHKU4r-CoV, cannot be neutralized by any of the eight MERS-CoV S RBD-targeting mAbs tested (Figure 2B and Table 1). The homology assessment of the RBD amino acids to A/EMC/2012 showed that the similarity of B/KNIH002 and C/HKU270 is >99%, while those of the other two merbecoviruses, BtCoV-422 (69.7%) and MjHKU4r-CoV (66.2%) were clearly low. These results suggest that the eight mAbs targeting MERS-CoV S RBD tested in this study can cross-react against MERS-CoV but not against merbecoviruses from bats and pangolins.

**Table 1:** Raw IC50 values of neutralization assay against five merbecoviruses tested.

	IC50 (50% inhibitory concentration, μg/ml)									
Antibody	D12	JC57-14	m336	MCA-1	CDC2-C2	MERS-4 V2	KNIH90-F1	REGN3051	G4	7D10
Virus										
A/EMC/2012	0.087	0.039	0.19	0.037	0.060	0.16	0.031	0.020	5.9	50
	0.077	0.023	0.20	0.031	0.050	0.28	0.037	0.012	6.4	50
	0.041	0.047	0.041	0.11	0.013	0.20	0.041	0.0074	12	50
Average	0.068	0.037	0.14	0.059	0.041	0.21	0.037	0.013	8.1	50
B/KNIH002	0.25	0.090	1.2	0.082	0.17	0.20	0.036	0.013	3.3	2.8
	0.18	0.043	1.2	0.060	0.30	0.76	0.057	0.013	6.3	2.4
	0.14	0.060	0.14	0.15	0.041	0.38	0.076	0.018	14	17
Average	0.19	0.064	0.85	0.096	0.17	0.45	0.056	0.015	7.7	7.5
C/HKU270	0.095	0.066	0.52	0.084	0.12	0.41	0.062	0.054	5.2	>50
	0.098	0.059	0.51	0.047	0.21	0.39	0.073	0.023	8.7	>50
	0.061	0.039	0.064	0.071	0.039	0.23	0.046	0.021	14	>50
Average	0.085	0.055	0.36	0.067	0.12	0.34	0.060	0.033	9.3	>50
BtCoV422	>5.0	>5.0	>5.0	>5.0	>5.0	>25	>5.0	>25	2.8	6.1
	>5.0	>5.0	>5.0	>5.0	>5.0	>25	>5.0	>25	4.3	35
	>5.0	>5.0	>5.0	>5.0	>5.0	>25	>5.0	>25	25	27
Average	>5.0	>5.0	>5.0	>5.0	>5.0	>25	>5.0	>25	11	23
MjHKU4r-CoV	>5.0	>5.0	>5.0	>5.0	>5.0	>25	>5.0	>25	>25	>50
	>5.0	>5.0	>5.0	>5.0	>5.0	>25	>5.0	>25	>25	>50
	>5.0	>5.0	>5.0	>5.0	>5.0	>25	>5.0	>25	>25	>50
Average	>5.0	>5.0	>5.0	>5.0	>5.0	>25	>5.0	>25	>25	>50

### V530L substitution contributes to the resistance of MERS-CoV B/KNIH002 to an mAb, m336

**Figure 3:**
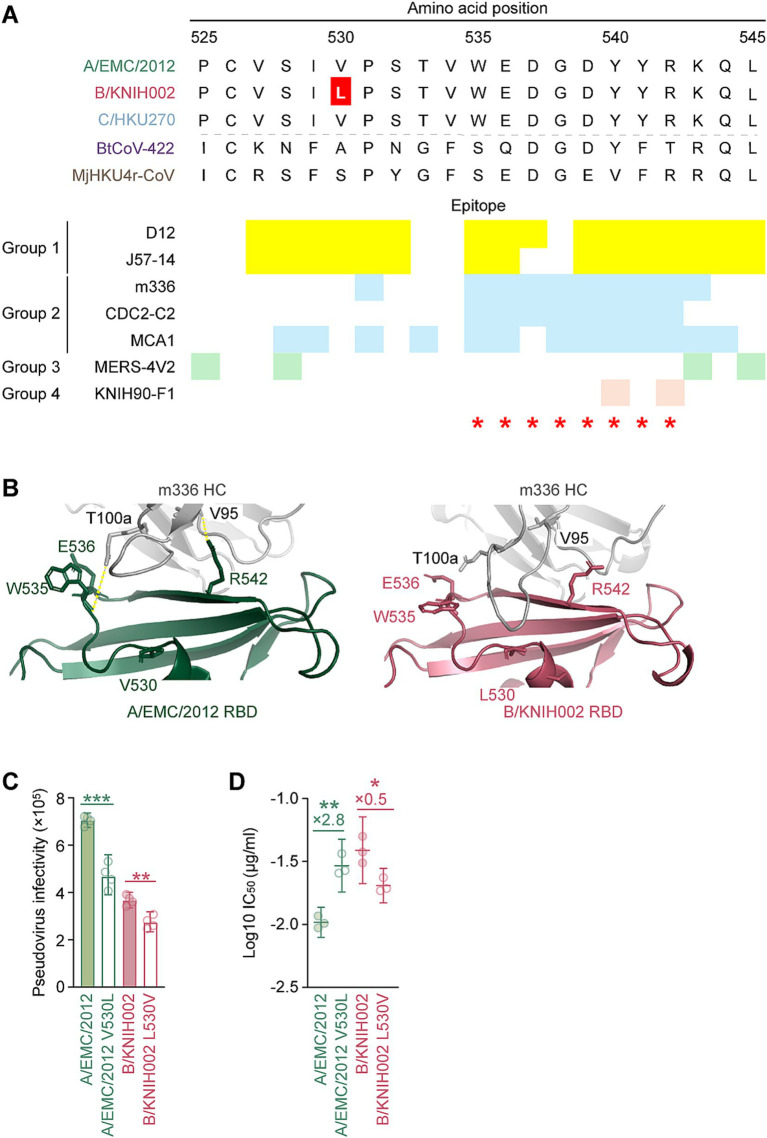
V530L substitution of MERS-CoV B/KNIH002 escaping from neutralization by m336. **(A)** Epitopes of the RBD-targeting mAbs in the alignment of the RBD amino acids sequences of three MERS-CoV strains and two merbecoviruses. The amino acids 525–545 sequences is truncated to show the epitopes of mAbs marked in different colors. The yellow, blue, green and pink represent the epitopes of mAbs from Group 1, Group 2, Group 3 and Group 4, respectively. The amino acid at position 530, highlighted with a red color, differs among MERS-CoV strains: L is present in B/KNIH002, while V is present in A/EMC/2012 and C/HKU270. Binding sites of DPP4 are indicated by red asterisks. **(B)** Structural insights of the mutation in B/KNIH002 RBD bound by m336. The interaction regions in the complex structure of m336 and the A/EMC/2012 RBD (PDB: 4XAK) (left) and the region in the complex structure of m336 and the B/KNIH002 RBD estimated by AlphaFold3 (right) are shown. In the structures, gray represents the m336 heavy chain, green represents A/EMC/2012 RBD, and pink represents B/KNIH002 RBD. Representative interactions between m336 and A/EMC/2012 RBD are shown as lines with yellow dots. **(C)** Pseudovirus infectivity. HOS-human DPP4/TMPRSS2 cells were infected with pseudoviruses bearing each S. The amount of input virus was adjusted to match the amount of HIV-1 p24 capsid protein. Assays were performed in quadruplicate. The presented data are expressed as the average ± SD of relative light unit (RLU). Each dot indicates the result of an individual replicate. Statistically significant differences (**p < 0.01; ***p < 0.001) versus parental S were determined by two-sided Student’s *t-*test. **(D)** Neutralization assay with m336 and MERS-CoV point mutants at position 530. A neutralization assay was performed using pseudoviruses with A/EMC/2012, B/KNIH002, A/EMC/2012 V530L and B/KNIH002 L530V S proteins, and the mAb, m336. Statistically significant differences (**p < 0.01; *p < 0.05) versus each parental S protein were determined by two-sided Wilcoxon signed-rank tests.

We found that B/KNIH002 was highly resistant to m336 compared to the other RBD-targeting mAbs (Figure 2B). To determine the key amino acid residues of B/KNIH002 that are responsible for the immune resistance to m336, the amino acid sequences in the epitope regions of the RBDs of the three MERS-CoV strains were compared. As shown in Figures 3A,B/KNIH002 harbored a unique amino acid substitution at the position 530 of the S protein: B/KNIH002 harbored leucine (L), while A/EMC/2012 and C/HKU270 harbored valine (V) at this position (Figure 3A). Importantly, the residue at position 530 is inside the epitopes that are targeted by mAbs from group 1 and adjoins the epitopes that are targeted by mAbs from group 2 (Figure 3A) (Jang et al., 2022; Xu et al., 2019).

To assess the effect of the amino acid residue at position 530 of MERS-CoV S on the interaction with m336, we first used AlphaFold3 to model the structure for analyzing the interface between m336 and RBD of B/KNIH002 (Figure 3B). The binding mode of the heavy chain (HC) of m336 and A/EMC/2012 S RBD in the crystal structure (PDB: 4XAK) shows that residue T100a of the m336 HC binds to residue W535 and residue V95 binds to R542 on the A/EMC/2012 RBD through hydrogen bonds (Figure 3B). AlphaFold3 shows the similar structure of A/EMC/2012 RBD in the complex with m336 to the crystal structure (PDB: 4XAK) with RMSD 0.60 Å. On the other hand, m336 CDRH3 forms a bit different loop structures in AlphaFold3 prediction as previous studies suggested that flexible regions are less accurate (Wee and Wei, 2024). However, the m336 CDRH3 in the AlphaFold3 structure maintained the disulfide bond between C98 and C100c and binding to the key residue, R542, of the epitope on A/EMC/2012 RBD (Supplementary Figure S1). Therefore, we believe that AlphaFold3 can detect the key interaction between m336 and B/KNIH002 as well. When the B/KNIH002 RBD is modeled with m336 by AlphaFold3, it is observed that m336 HC approaches the mutated residue V530L on the B/KNIH002 RBD and is no longer bound to either W535 or R542 on the B/KNIH002 RBD (Figure 3B and Supplementary Figure S2). In detail, three remarkable alterations are observed in the local structure of B/KNIH002 RBD: line-symmetric rotation of W535 sidechain and R542 sidechain, and outward shift of E536 sidechain (Supplementary Figure S2). In addition, the loop region where W535 is located is slightly uplifted with the continuing β7 strand probably due to the longer side chain of leucine than that of valine (Supplementary Figure S2). This stretched epitope appears to have less impact on the interaction with two or more complementarity-determining regions (CDRs) of mAbs (Figures 2B and Supplementary Figure S3). Conversely, the steric change seems to be beyond the capability of mAbs binding to the most part of the epitope by a single CDR including m336 and CDC-C2 (Figure 2B and Supplementary Figure S3C).

Next, we investigated the effect of the V530L substitution on pseudovirus phenotypes using two S pseudovirus derivatives, A/EMC/2012 carrying V530L and B/KNIH002 carrying L530V. As shown in Figure 3C, the V530L substitution significantly decreased the pseudovirus infectivity of A/EMC/2012. Additionally, the L530V substitution also significantly decreased the pseudovirus infectivity of B/KNIH002 (Figure 3C). These results suggest that the effect of the amino acid residue at position 530 on pseudovirus infectivity is dependent on the S protein backbone.

We then assessed whether the amino acid residue positioned at 530 is responsible for the sensitivity to m336 by neutralization assay. We found that the V530L substitution significantly increased the 50% inhibitory concentration (IC_50_) value of m336 against A/EMC/2012 (2.8-fold) (Figure 3D and Table 2), suggesting that the V530L substitution confers resistance to m336-mediated neutralization. In sharp contrast, the L530V substitution in B/KNIH002 significantly (0.5-fold) decreased the IC_50_ value (Figure 3D and Table 2), suggesting that the L530V substitution renders B/KNIH002 more sensitive to m336. Altogether, these results suggest that residue L530 in B/KNIH002 is responsible for the resistance to m336-mediated neutralization. Notably, the V530L substitution was detected only in three strains of MERS-CoV clade B emerged during the outbreak in South Korea (Supplementary Table S1).

**Table 2:** Raw IC50 values of neutralization assay using m336 against the residue 530 derivatives.

Virus	Average (standard deviation)
A/EMC/2012	0.010 (0.0012)
A/EMC/2012 V530L	0.030 (0.0060)
B/KNIH002	0.040 (0.010)
B/KNIH002 L530V	0.020 (0.0030)

### Different neutralization spectrum of the mAbs targeting NTD and S2

**Figure 4:**
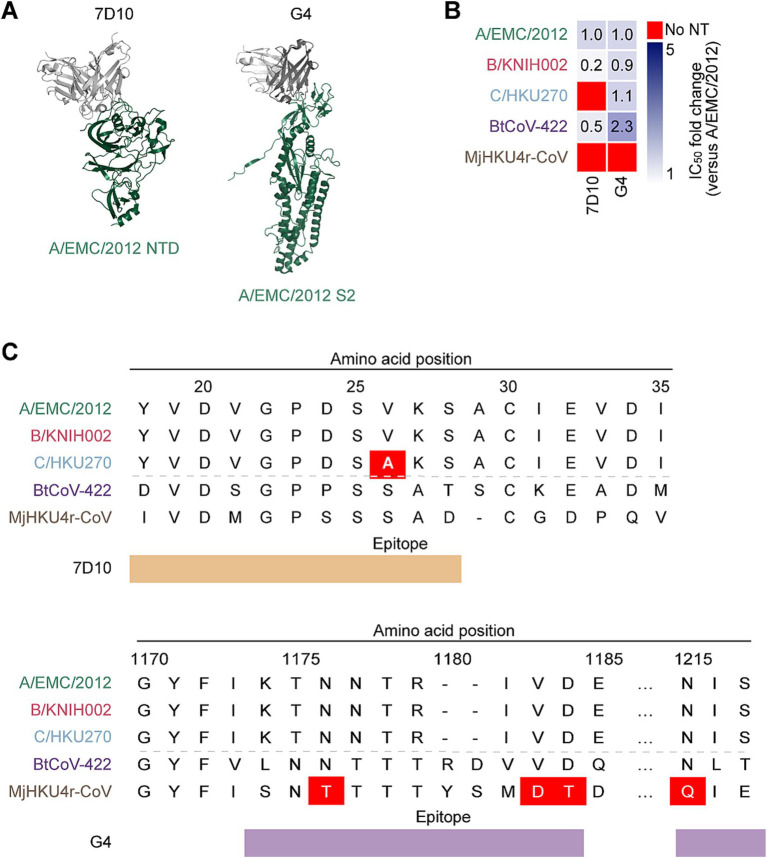
Neutralization spectrum and epitopes of the anti NTD mAb and the anti-S2 mAb. **(A)** Binding modes of MERS-CoV neutralizing mAbs, 7D10 (PDB: 6 J11) and G4 (PDB: 5WQM), targeting NTD and S2 subunit of A/EMC/2012 RBD. The RBD is colored in green and antibodies in gray. **(B)** Neutralization breadth of the non-RBD targeting mAbs. The heatmap displays the fold change in 50% inhibitory concentration (IC_50_) values, divided by an IC_50_ against A/EMC/2012. The color scheme shows the IC_50_ fold change against 3 clades of MERS-CoVs in addition to BtCoV-422 and MjHKU4r-CoV as the intensity of the blue color in the heatmap. The darkest blue indicates either the fold change more than 6 or the mAb failed to reach IC_50_ at the highest concentration tested. The red indicates no neutralization (no NT). **(C)** The epitopes of 7D10 and G4 in the alignment of the NTD and S2 amino acids sequences of three MERS-CoV strains together with BtCoV-422 and MjHKU4r-CoV. The NTD ranging amino acid 18 to amino acid 35 is shown the upper table with the epitope recognized by 7D10 marked in brown. The amino acid at position 26 of C/HKU270, highlighted with a red color, differs from A/EMC/2012 and B/KNIH002. The S2 subunit ranging amino acid 1,170 to amino acid 1,217 is shown the lower table with the epitope recognized by G4 marked in violet. The amino acid at position 1,176, 1,183–4 and 1,215 of MjHKU4r-CoV differ from MERS-CoVs and BtCoV-422.

In addition to the mAbs targeting RBD, we tested neutralization ability of two non-RBD targeting mAbs against merbecoviruses: 7D10 targets the NTD and G4 targets the S2 subunit of MERS-CoV S protein (Pallesen et al., 2017; Zhou et al., 2019) (Figure 4A). To explore the cross-reactivity of these mAbs, we performed a neutralization assay with the pseudoviruses of the five merbecoviruses. We found that 7D10 neutralized A/EMC/2012 and B/KNIH002 but failed to neutralize activity against C/HKU270. A previous study reported that the V26A substitution is located in the epitope of 7D10, and leads to resistance to neutralization by 7D10 (Zhou et al., 2019). Consistently, we detected the V26A substitution in the S protein of C/HKU270 (Figure 4C). Altogether, these observations suggest that residue A26 is responsible to the resistance of C/HKU270 to 7D10 (Figure 4B).

In contrast to C/HKU270, B/KNIH002 exhibited a higher sensitivity to 7D10 compared to A/EMC/2012, with average IC_50_ values of 7.48 μg/mL and 50.0 μg/mL, respectively. (Figure 4B and Table 1). Notably, 7D10 cross-neutralized the bat CoV BtCoV-422 more efficiently than A/EMC/2012, with an average IC_50_ value of 22.67 μg/mL. On the other hand, MjHKU4r-CoV cannot be cross-neutralized by 7D10 (Figure 4B and Table 1). Corresponding to previous reports (Zhou et al., 2019; Zhang et al., 2022), our results showed that C/HKU270 obtained neutralization resistance by a single mutation of V26A on the NTD. In the N-terminal region of the NTD, there are two different amino acids between BtCoV-422 and MjHKU4r-CoV at the second amino acid positions both sides from V26A. Zhou revealed that V24A induced complete loss of binding to 7D10, but S28A was less effective (Zhou et al., 2019). Based on this information, D24S seems to have more impact than D24P on the binding of 7D10 in hydrophobic circumstance surrounding D24, because neither a hydrophobic interaction nor a hydrogen bond can be formed between S24 and Y117. Furthermore, the deletion of A29 and S28D contribute to the neutralization resistance of MjHKU4r-CoV against 7D10 due to steric changes.

In the case of G4 that targets the S2 subunit of S protein, the neutralizing ability of this antibody against three MERS-CoV strains was less potent compared to the eight RBD-targeting mAbs (Table 1). However, it was noted that G4 cross-neutralizes BtCoV-422 with higher neutralization potency than 7D10, with an average IC_50_ value of 10.69 μg/mL (Table 1). Although non-RBD mAbs tested potentially neutralized BtCoV-422, none of the mAbs tested in this study were able to cross-neutralize MjHKU4r-CoV (Table 1).

## Discussion

In this study, we demonstrated the distinct cross-neutralization abilities of previously isolated anti-MERS-CoV mAbs, including eight RBD-targeting and two non-RBD-targeting mAbs, against pseudoviruses representing five DPP4-using merbecovirus groups (Figure 1): MERS-CoVs from the three major clades, and the MERS-related bat CoV BtCoV-422 and the HKU4-related pangolin CoV MjHKU4r-CoV. Notably, the RBD-targeting mAb, m336, exhibited significantly reduced neutralization against B/KNIH002 compared to A/EMC/2012, primarily due to the V530L substitution found in B/KNIH002. Interestingly, this V530L substitution was detected exclusively in three South Korean strains from clade B (Supplementary Table S1). This observation strongly suggests that the V530L substitution emerged during the MERS outbreak in South Korea in 2015. However, the fact that the V530L substitution was not predominant in the South Korean outbreak suggests that this mutation was not beneficial for efficient spread of MERS-CoV. Moreover, the impact of this substitution on receptor binding and viral fitness remains undetermined. According to a previous study (Wang et al., 2013), V530 does not directly interacts with DPP4. While the infectivity of A/EMC/2012 V530L compared to A/EMC/2012 WT was decreased (Figure 3C), deep mutational scanning revealed that V530L lead to a slight increase of MERS-CoV binding to DPP4,^1^ potentially through an indirect interaction. This suggests that this mutation has an important role in efficient binding and may contribute to an increased viral fitness. However, due to the low number of isolated MERS-CoVs possessing V530L, it is uncertain if this mutation has a broad and significant effect on MERS-CoV evolution.

The epitope of m336 and other Group 2 mAbs are located at the region of the RBD close to an amino acid at position 530 overlapped with the epitopes of Group 1 mAbs and the DPP4 binding site (Figure 3A). Cross-clade neutralization analysis showed that two of the Group 2 mAbs possesses significant fold changes on the IC_50_ values of A/EMC/2012 and B/KNIH002. For instance, m336 showed the highest fold change of 5.7 and CDC2-C2 showed the second highest fold change of 4.1, while the fold changes of other Group 1 and Group 2 mAbs were less than 3 folds (Figures 2B, 3A). Structural model reveals that V530L interacts with Group 1 and Group 2 epitopes interfering with the binding of a single CDR of m336 and CDC2-C2 (Figure 3B, Supplementary Figures S2, S3C), though Valine and Leucine are similar chemical properties of nonpolar, hydrophobic amino acids. This suggests either partial or total loss of bindings of m336 HC to the RBD including the high-affinity interaction by a hydrogen bond between V95 and R542, and R100e and D539 (Ying et al., 2015) resulting in the reduced neutralizing ability of m336 against B/KNIH002 (Figure 3B). Furthermore, since m336 was derived from a naïve human phage-displayed antibody library, m336 possibly is more vulnerable to the mutation of B/KNIH002 obtained during outbreak compared to other Group 2 mAbs, CDC2-C2 and MCA1 which is derived from human survivors of MERS-CoV infection (Chen et al., 2017; Xu et al., 2019).

This study also highlights the broad but low-potency neutralization of non-RBD targeting mAbs. The anti-NTD mAb, 7D10, exhibits cross-neutralization to the bat CoV BtCoV-422, but its neutralization efficiency is very weak against A/EMC/2012 and ineffective to C/HKU270 as well as MjHKU4r-CoV. Sequence analysis shows V26A substitution is a distinct mutation on 7D10 epitope of C/HKU270 among MERS-CoVs potentially attributing to humoral immune evasion (Figures 4B,C) (Zhou et al., 2019). However, in the cases of BtCoV-422 and MjHKU4r-CoV, there are many differences of amino acids in the NTD (Figure 4C) and further study is required to explore the key residues contributing to the difference of susceptibility to 7D10 neutralization. Another tested non-RBD targeting mAb, G4, showed the widest neutralization breadth, although the IC_50_ value was relatively higher than anti-RBD mAbs (Figure 4B and Table 1). These observations suggest that G4-like mAbs may be a candidate for broadly neutralizing mAb against a wide range of merbecoviruses. Recently, machine learning-based methods to improve mAb affinity has been developed (Hie et al., 2024). Therefore, improving the breadth and neutralizing activity by using machine learning-based methods may be a strategy for preparing mAbs that can be used for therapeutics of merbecovirus infection in the future.

To our knowledge, almost all neutralizing mAbs against MERS-CoV have been evaluated using HCoV-EMC/2012 (clade A) as a reference virus. However, clade A has no recent reports of circulation, and clade B is currently circulating in the human population. As shown in this study, all mAbs against MERS-CoV clade A do not necessarily cross-react against other MERS-CoV clades. Moreover, evaluation of the cross-reactivity of each neutralizing mAb would be important to consider the future risk caused by other merbecoviruses.

While clade B MERS-CoV is the only clade currently known to cause outbreaks in humans, clade C viruses are increasingly recognized as a potential zoonotic threat (Karani et al., 2025). Individuals working closely with camels, such as herders, traders, and abattoir workers, experience frequent, high-risk exposures that may allow clade C viruses to cross the species barrier (Kamau et al., 2019). Although these infections may be asymptomatic or go undiagnosed due to limited surveillance, recent serologic data suggest human exposure does occur (Ogoti et al., 2024). These findings emphasize the need to monitor clade C viruses and the human populations in contact with them, especially in settings where human–camel interfaces are intensive, and biosecurity is limited.

Here we showed the sensitivity of five merbecoviruses to ten neutralizing mAbs, but this study includes some limitation. First, since no antibody was available to detect the S proteins of the merbecoviruses tested in this study, the expression levels and virion incorporation efficiency of each S protein were not quantified. It is possible that some anti-MERS-CoV S antibodies may cross-react with other merbecovirus S proteins. However, there is no guarantee that this detection level is quantitative. It should also be noted that the results of this study are based on a lentivirus-based pseudovirus system. In addition to the multi-epitope antibody combination strategy, these results may be reevaluated using live viruses.

In summary, here we demonstrated the potency of RBD-targeting mAbs against MERS-CoV strains from different clades and the neutralizing breadth of non-RBD-targeting mAbs against other merbecoviruses. One of the advantages of therapeutic or prophylactic use of mAbs is the reduced adverse events caused by off-target bindings. Conversely, the disadvantage is neutralization evasion induced during virus spread, sometimes by a single substitution as observed in this study. Combining mAbs targeting different epitopes could be protective in zoonotic transmission and can prevent human-to-human transmission.

## Materials and methods

### Accession numbers

The gene sequences for S proteins used in this study were achieved from the GenBank. The accession numbers are JX869059 (MERS-CoV EMC/2012), KT029139 (MERS-CoV KNIH002), KJ477103.2 (MERS-CoV HKU270), MG021452 (BtCoV/Ii/GD/2014–422) and OQ786862.1 (MjHKU4r-CoV).

### Data acquisition and phylogenetic analysis

The genome sequences of all merbecoviruses were obtained as of August 30, 2024, from the NCBI Virus database^2^ using the search query: merbecovirus, taxid: 2509494. Only sequences classified as ‘complete’ under the Nucleotide Completeness category were retrieved. Representative sequences from each group were selected for phylogenetic analysis based on the classification of merbecovirus groups as previously described (Tolentino et al., 2024). To account for the uneven representation of MERS-CoV sequences in the database, we selected two complete genomes for clade A, including the NCBI reference genome (HCoV-EMC/2012, accession: NC_019843.3), two complete genomes for clade C, and two complete genomes from each lineage of clade B. To expand the dataset for infectivity and neutralization studies, all available genome sequences linked to the 2015 MERS-CoV outbreak in South Korea—the largest known outbreak outside the Arabian Peninsula (Korea Centers for Disease C, Prevention, 2015)—were included. Additionally, two bat coronavirus genomes identified as merbecoviruses (isolates PREDICT/PDF-2180 and PnNL2018B, with accessions NC_034440.1 and OQ405399.1, respectively) were included, despite not being classified under the merbecovirus subgenus in the database. To root the merbecovirus phylogeny, we incorporated four representative betacoronavirus genomes: Sarbecovirus (Wuhan-Hu-1, NC_045512.2), Nobecovirus (HKU9, EF065513.1), Hibecovirus (Hp-betacoronavirus/Zhejiang2013, KF636752.1), and Embecovirus (HKU1, NC_006577.2).

To determine the evolutionary relationships of the selected sequences, maximum likelihood tree was constructed. Complete genome sequences were aligned through MAFFT v.7.520 (Katoh and Standley, 2013) using default settings. An in-house Python script was then employed to process the aligned sequences, replacing any characters outside of ‘ATGCN-’ with ‘N’. The cleaned sequences were subsequently analyzed in IQTree v.2.2.2.6 (Minh et al., 2020) using the general-time reversible (GTR) nucleotide substitution model. Node support was assessed using ultrafast bootstrap (Minh et al., 2013) performed over 1,000 iterations.

### Plasmid construction

Oligonucleotides coding for the codon-optimized MERS-CoV S proteins of A/EMC/2012 (GenBank accession no. JX869059), B/KNIH002 (GenBank accession no. KT029139), C/HKU270 (GenBank accession no. KJ477103.2); and the codon-optimized MERSr-CoV S proteins of BtCoV-422 (GenBank accession no. MG021452) and MjHKU4r-CoV (GenBank accession no. OQ786862.1) were synthesized by a gene synthesis service (Fasmac). The oligonucleotides for merbecovirus S proteins were amplified by polymerase chain reaction (PCR) using primers listed in Supplementary Table S2. The resulting PCR fragment was subcloned into the KpnI-NotI site of the pCAGGS vector (Niwa et al., 1991) using In-Fusion HD Cloning Kit (Takara, Cat# Z9650N). Plasmids expressing DPP4 was prepared in a previous study (Barabona et al., 2024).

The point mutations of V530L and L530V were induced into the pCAGGS-A/EMC/2012 and pCAGGS-B/KNIH002 by site-directed overlap extension PCR using primers listed in Supplementary Table S2, respectively. To construct the plasmids expressing anti-MERS-CoV monoclonal antibodies (mAbs) (m336, KNIH90-F1, MERS-4 V2, D12, CDC2-C2, JC57-14, MCA1, REGN3051, 7D10, G4), the sequences of the variable regions of these antibodies were obtained from PDB Database^3^ and were artificially synthesized (Fasmac). The obtained DNA fragments coding the variable regions of the heavy and light chains were cloned into the pCAGGS vector containing the sequences of the human immunoglobulin 1 and kappa constant region correspondingly [kindly provided by Dr. Hisashi Arase (Osaka University, Japan)]. Nucleotide sequences were determined by DNA sequencing services (Eurofins) and analyzed by Snapgene software v.7.2.1.^4^

### Cell culture

LentiX-293 T cells (Takara, Cat# 632180) were cultured in Dulbecco’s modified Eagle’s medium (DMEM) (high glucose) (Wako, Cat# 044–29,765) containing 10% fetal bovine serum (Sigma-Aldrich, Cat# 172012-500ML), 100 U/mL penicillin, and 100 μg/mL streptomycin (Sigma-Aldrich, Cat# P4333-100ML). HOS-hDPP4/TMPRSS2 cells were prepared in the previous study (Barabona et al., 2024) and were maintained in high-glucose DMEM supplemented with the same components, along with 0.5 μg/mL puromycin (InvivoGen, Cat# ant-pr-1) and 1 mg/mL G418 (Nacalai Tesque, Cat# 09380–86). All cell lines were authenticated and tested for mycoplasma contamination.

### Pseudovirus preparation

Pseudovirus infection (Figures 2B, 3C) was performed as previously described (Suzuki et al., 2022; Motozono et al., 2021; Meng et al., 2022; Ferreira et al., 2021; Uriu et al., 2021). Briefly, lentivirus (HIV-1)-based, luciferase-expressing reporter viruses were pseudotyped with the merbecoviruses S. One prior day of transfection, the LentiX-293 T cells were seeded at a density of 2 × 10^6^ cells. The LentiX-293 T cells were cotransfected with 1 μg psPAX2-IN/HiBiT a packaging plasmid encoding the HiBiT-tag-fused integrase (Ozono et al., 2021), 1 μg pWPI-Luc2 a reporter plasmid encoding a firefly luciferase gene (Ozono et al., 2020) and 500 ng plasmids expressing representative merbecoviruses S using TransIT-293 transfection reagent (Mirus, Cat# MIR2704) according to the manufacturer’s protocol. Two days post-transfection, the culture supernatants were harvested and filtrated. The amount of produced pseudovirus particles was quantified by the HiBiT assay using Nano Glo HiBiT lytic detection system (Promega, Cat# N3040) as previously described (Ozono et al., 2020). In this system, HiBiT peptide is produced with HIV-1 integrase and forms NanoLuc luciferase with LgBiT, which is supplemented with substrates. In each pseudovirus particle, the detected HiBiT value is correlated with the amount of the pseudovirus capsid protein, HIV-1 p24 protein (Ozono et al., 2020). Therefore, we calculated the amount of HIV-1 p24 capsid protein based on the HiBiT value measured, according to the previous paper (Ozono et al., 2020). To measure viral infectivity, the same amount of pseudovirus, adjusted to the same HIV-1 p24 capsid protein level was inoculated into HOS-hDPP4/TMPRSS2 cells. At 2 days postinfection, the infected cells were lysed with a Bright-Glo luciferase assay system (Promega, Cat# E2620), and the luminescent signal produced by firefly luciferase reaction was measured using a GloMax explorer multimode microplate reader 3,500 (Promega). The pseudoviruses were stored at −80°C until use.

### Monoclonal antibody production and purification

m336, KNIH90-F1, MERS-4 V2, D12, CDC2-C2, JC57-14, MCA1, REGN3051, 7D10, G4 were prepared as previously described (Yamasoba et al., 2022; Yamasoba et al., 2022). Briefly, the pCAGGS vectors containing the sequences encoding the immunoglobulin heavy and light chains were cotransfected into LentiX-293 T cells using PEI MAX Transfection Reagent (Polysciences, Cat# 24765–1). At 48 h post transfection, the cell culture supernatants were harvested, and the antibodies were purified using NAb protein A plus spin kit (Thermo Fisher Scientific, Cat# 89948) according to the manufacturer’s protocol.

### Neutralization assay

Neutralization assays were performed as previously described (Yamasoba et al., 2022). Briefly, the mAbs were diluted by 5-fold serial dilution (up to 15,625-fold) from the initial concentration: 5 μg/mL for m336, MCA1, CDC2-C2, and D12; 25 μg/mL for JC57-14, MERS-4 V2, KNIH90-F1, REGN3051, and G4; and 50 μg/mL for 7D10. Then, the diluted mAbs were incubated with the merbecovirus S pseudoviruses (counting~150,000 relative light units/15 μL) at 37°C for 1 h. Pseudoviruses without antibodies were included as controls. Then, 20 μL mixture of a pseudovirus and a mAb was added to pre-seeded HOS-hDPP4/TMPRSS2 cells (10,000,000 cells/100 μL) in a 96-well white plate. At 2 days post infection, the infected cells were lysed with a Bright-Glo Luciferase Assay System (Promega, Cat# E2620), and the luminescent signal was measured using a GloMax explorer multimode microplate reader 3,500 (Promega). The assay of each antibody was performed in triplicate, and the 50% inhibitory concentration (IC_50_) was calculated using Prism 10 software v.10.4.0 (GraphPad Software).

### Protein structure model

The costructure of B/KNIH002 S RBD and the antibody m336 was predicted using AlphaFold3 (Abramson et al., 2024). Evaluation of the models were performed using pLDDT scores, pTM scores and ipTM scores then the best model for the costructure was selected (Abramson et al., 2024). All protein structural analyses were performed using the PyMOL molecular graphics system v.3.0.0 (Schrödinger).

### Epitope analysis

AppA server (Nguyen et al., 2019) is used to determine binding interface between antibody and RBDs in PDB files (PDB:4ZPT for D12, PDB:6C6Y for JC57-14, PDB:4XAK for m336, PDB:6C6Z for CDC-C2, PDB:5GMQ for MCA1, PDB:5YY5 for MERS-4 V2, PDB:7COE for KNIH90-F1, PDB:6 J11 for 7D10, 5WQM for G4). Mol* 3D Viewer is utilized for binding analysis (Sehnal et al., 2021). The epitopes are depicted in amino acid sequences of the five merbecoviruses aligned by by SnapGene software v6.2.50 (See Text footnote 4).

## Data Availability

The datasets presented in this study can be found in online repositories. The names of the repository/repositories and accession number(s) can be found in the article/Supplementary material.

## Footnotes

^1^ https://github.com/jbloomlab/MERS-PDF2180-RBD_DMS

^2^ https://www.ncbi.nlm.nih.gov/labs/virus/vssi/#/

^3^ https://www.rcsb.org

^4^ www.snapgene.com
