# Supplementary figures and images for: Cross-neutralization ability of anti-MERS-CoV monoclonal antibodies against a variety of merbecoviruses
Source: Front Microbiol. 2025 Jul 16;16:1593095. doi: 10.3389/fmicb.2025.1593095 (PMC12307460; doi:10.3389/fmicb.2025.1593095)

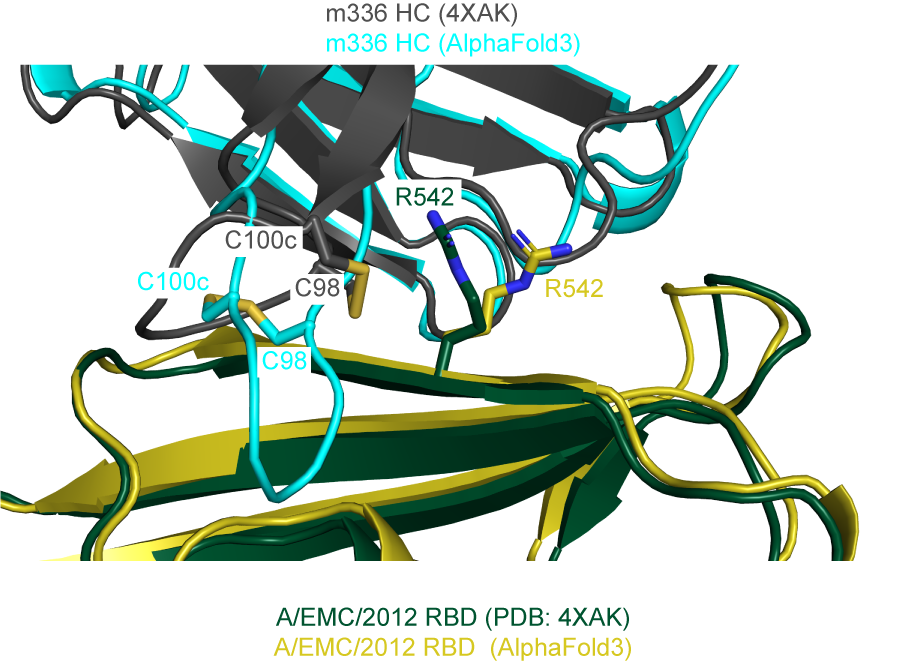

Supplement: Supplementary file 3 [file Image_1.tif]

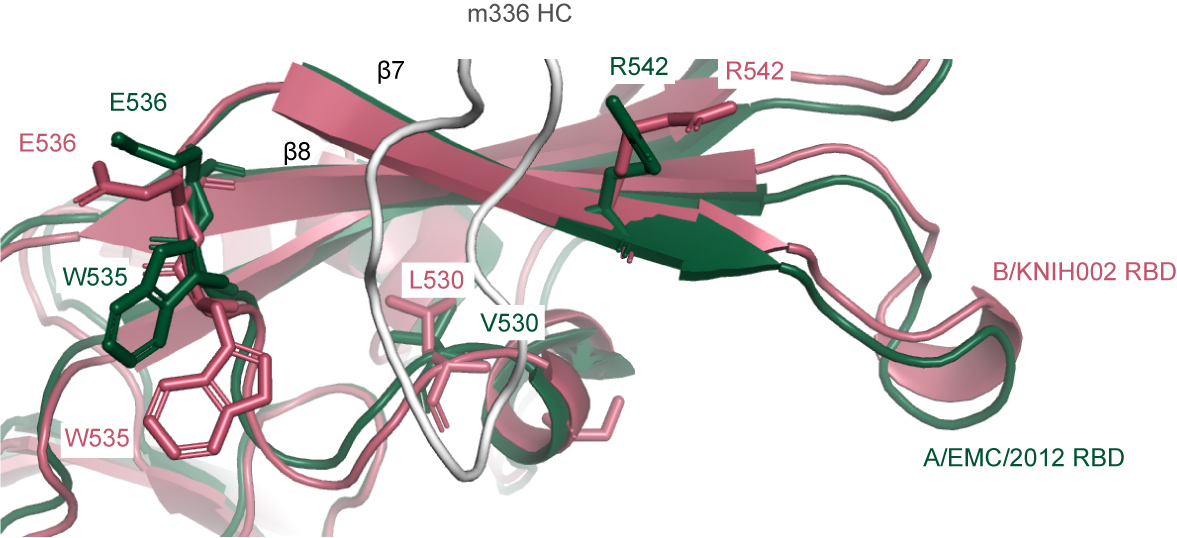

Supplement: Supplementary file 4 [file Image_2.tif]

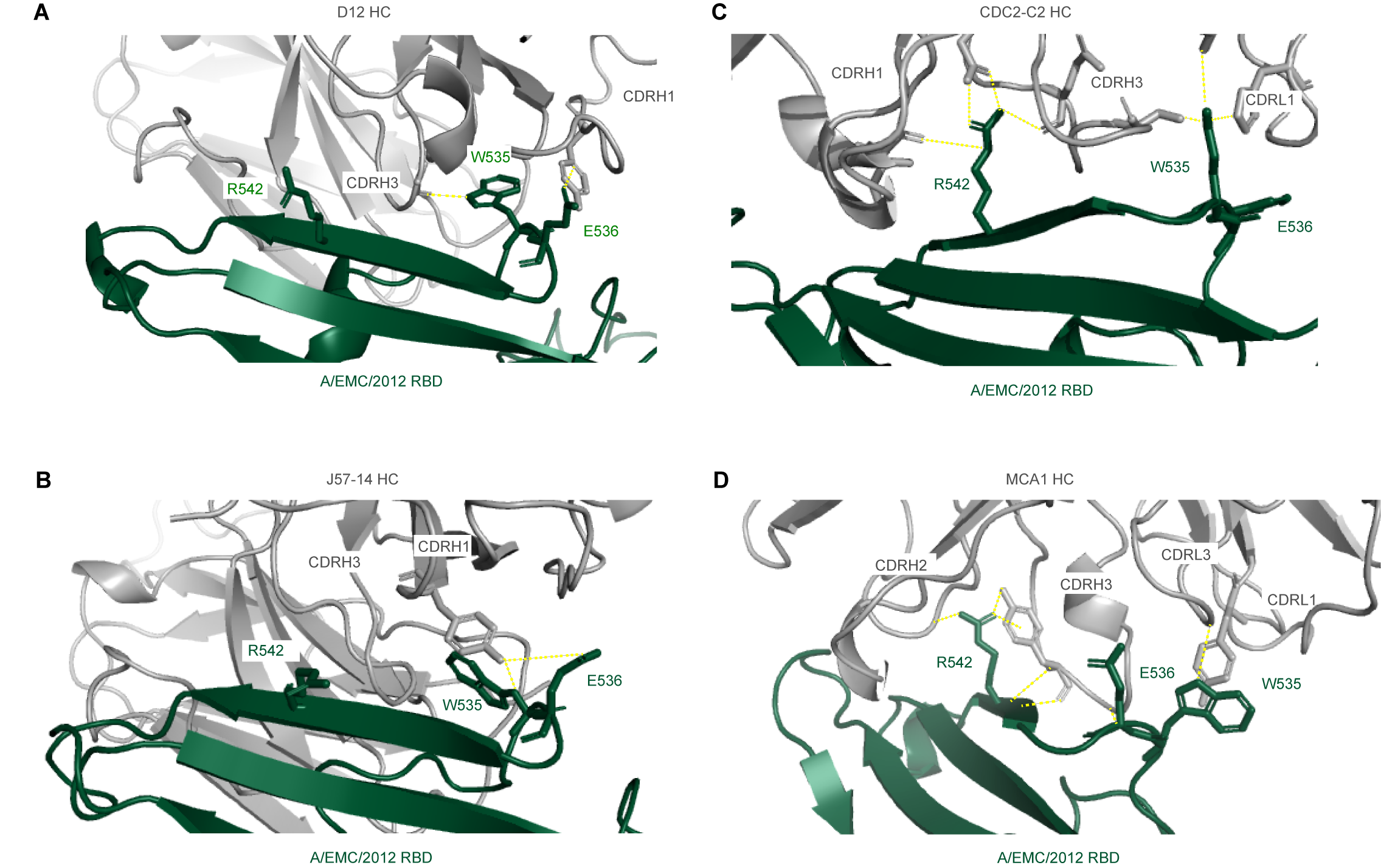

Supplement: Supplementary file 5 [file Image_3.tif]
